# Effect of fissure angle on energy evolution and failure characteristics of fractured rock under uniaxial cyclic loading

**DOI:** 10.1038/s41598-022-26091-4

**Published:** 2023-02-15

**Authors:** Yongqiang Zhao, Quanshen Li, Kai Zhang, Yingming Yang, Xuebin Gu

**Affiliations:** 1 Beijing Low-Carbon Clean Energy Research Institute National Energy Group State Key Laboratory of Coal Mining Water Resources Protection and Utilization, Beijing, 102209 China; 2 National Energy Investment Group Co, Beijing, 100011 China; 3 China Shenhua Energy Company Limited, Beijing, 100011 China; 4 Shendong Coal Group Co.,Ltd, Shenmu, 719315 China; 5 College of Energy and Mining Engineering, Shandong University of Science and Technology, Qingdao, 266590 China

**Keywords:** Civil engineering, Mechanical engineering

## Abstract

To study the influence of fissure angle on the rock damage process and energy evolution characteristics, uniaxial cyclic loading and unloading tests were conducted on fractured rock specimens with different prefabricated fissure angles. The stress–strain curves, mechanical properties, and failure characteristics were analyzed. Subsequently, the energy evolution characteristics and failure mechanisms were investigated. The results showed that the stress–strain curves of fractured specimens fluctuated in the pre-peak phase and rapidly declined in post-peak phase. The peak stresses and strains of fractured specimens initially decreased and then increased with an increase in the fissure angle, whereas the elastic modulus first increased and then decreased. With an increase in the fissure angle, specimen failure changed from shear damage to tensile damage. The input, elastic, and dissipation energies of fractured specimens non-linearly increased with an increase in cyclic loading and unloading. As the number of cycles increased, the energy density decreased in segments with an increase in the fissure angle, and there was a rapid increase in the dissipation energy density before failure occurred. The results can provide a reference for the study of fractured rock failures and their prevention and control design in the field.

## Introduction

There are numerous defects such as fractures and pores inside the rock, and the mechanical responses of these defects under different stress conditions determine the performance of the macroscopic mechanical properties of rocks^1–3^. Crack initiation, expansion, and macroscopic crack formation correspond to different stages of damage in rocks, and failure occurs when the damage within the rock reaches its load-bearing limit. Therefore, it is important to study the mechanical properties and failure mechanisms of fractured rock masses to guide the development of field projects^4,5^.

Numerous experimental studies have been conducted on rocks that contain prefabricated fractures. The earliest research work was the uniaxial compression test on a single fractured specimen conducted by BOMBOLAKIS in 1968^6^. To gain more insight into the influence of prefabricated fractures on rock strength, deformation, and damage modes, scholars have systematically investigated the combination of form^7–9^, geometric parameters^10–12^, and the number of prefabricated fractures^13^. In addition, the site rock is mostly in a complex stress state, and the study of stress conditions is of great value for the crack extension of fracture specimens. Scholars have investigated the effects of lateral^14^ and confining pressures^15,16^ on the mechanical behavior and crack expansion patterns of prefabricated fractured rock samples to further reveal the damage mechanism of prefabricated fractured rocks. In addition, scholars have obtained the damage characteristics during rock destabilization by monitoring the fractured rock damage process using acoustic emission^17^, infrared cameras^18^, digital image correlation methods^19^, and CT^20^.

Many studies have shown that engineering rocks are often subjected to repeated loading and unloading of external stresses; thus, it is important to study the damage mechanisms of rocks under cyclic loading and unloading conditions^21–23^. Numerous studies have been conducted on the strength characteristics and deformation damage mechanisms of rocks under cyclic loading and unloading conditions^24^. Rock damage is a process of energy input, accumulation, dissipation, and release. The study of the energy evolution pattern of rocks under cyclic loading is important for revealing their damage mechanisms. Scholars have studied the energy evolution process during cyclic loading and unloading and proposed relevant damage evaluation metrics^25,26^. In summary, most studies have focused on the energy evolution characteristics of intact rocks only^27–30^, i.e., those of fractured rocks are rarely investigated, particularly under the cyclic loading and unloading conditions. Therefore, it is important to study the energy evolution laws of fractured rocks under the influence of the fissure angle.

In this study, uniaxial cyclic loading and unloading tests were conducted on precast fractured sandstone specimens with different fissure angles. The effects of the fissure angle on the basic mechanical properties and failure characteristics of the specimens were investigated. The energy evolution processes of different fracture dips during cyclic loading were analyzed. Based on this, the failure mechanism of fractured rocks is discussed. The research results can provide a reference for revealing the damage mechanism of fractured surrounding rocks at sites and for proposing targeted measures.

## Experimental setup

### Specimen preparation

**Figure 1:**
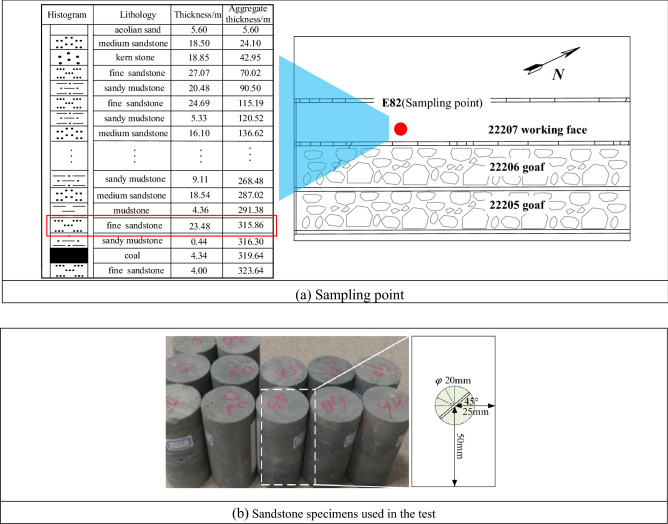
Specimen preparation.

The specimens used in the test were sandstone specimens obtained from the Buertai coal mine in the Shendong mining area in western China, and the sampling point is shown in Fig. 1a. First, the large sandstone specimens were transported to the laboratory for processing. Cutters and grinders were used to process cylindrical specimens 100 mm in height and 50 mm in diameter, ensuring that the non-parallelism of the two end faces was less than 0.05%, as shown in Fig. 1. Subsequently, ultrasonic tests were performed to select specimens with similar wave velocities and densities for the subsequent test. Finally, prefabricated fractured specimens were fabricated using wire-cutting equipment. The fissure length was 10 mm, width was 1 mm, and dip angles were 0°, 30°, 45°, 60°, and 90°, and three specimens of each type were made. The numbering according to the angle and number of specimens, for example, SY-30-1, indicates the first specimen with a 30° inclination. The basic mechanical parameters of the sandstone specimens are presented in Table 1.

**Table 1:** Basic mechanical parameters of the sandstone specimens.

Number	Density/g cm^−3^	Uniaxial compression strength/MPa	Elastic modulus/GPa	Uniaxial tensile strength/MPa
SY-1	2.37	30.12	2.47	6.57
SY-2	2.21	27.66	2.58	7.48
SY-3	2.45	28.00	1.84	7.11
Average value	2.34	28.59	2.30	7.05

### Experimental system and test scheme

**Figure 2:**
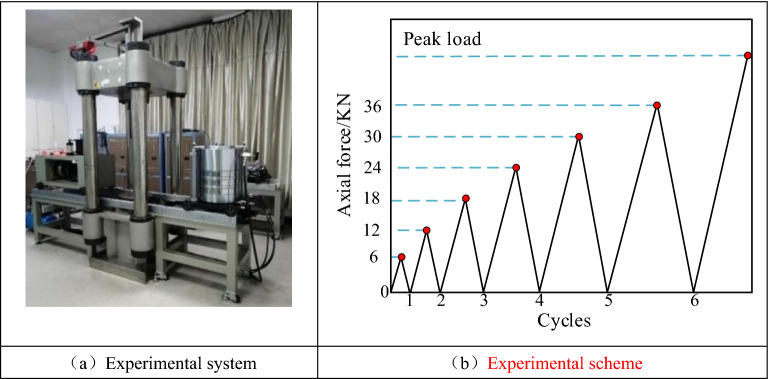
Experimental system and cyclic loading scheme.

The loading equipment was an RLJW-2000 rock mechanics testing machine, which provided a maximum loading force of 2000 kN and automatically recorded the stress and strain data, as shown in Fig. 2. The test was controlled by displacement loading, and the stress and deformation data were recorded using stress and displacement transducers that came with the test machine.

A scheme of the uniaxial cyclic loading and unloading tests is shown in Fig. 2b. The test process used the displacement control method, and the loading and unloading rate was 0.25 mm/min, with 6 kN as a cycle. Each load was incremented by 3 kN and then unloaded to 0.3 kN. That is, the load was increased from 0 → 6 kN → 0.3 kN → 12 kN → 0.3 kN → 18 kN → 0.3 kN → 24 kN …… step by step and stopped when the cycle reaches 36 kN. The specimen was then loaded at a loading rate of 0.25 mm/min until it was damaged.

## Test results

### Stress–strain curves

**Figure 3:**
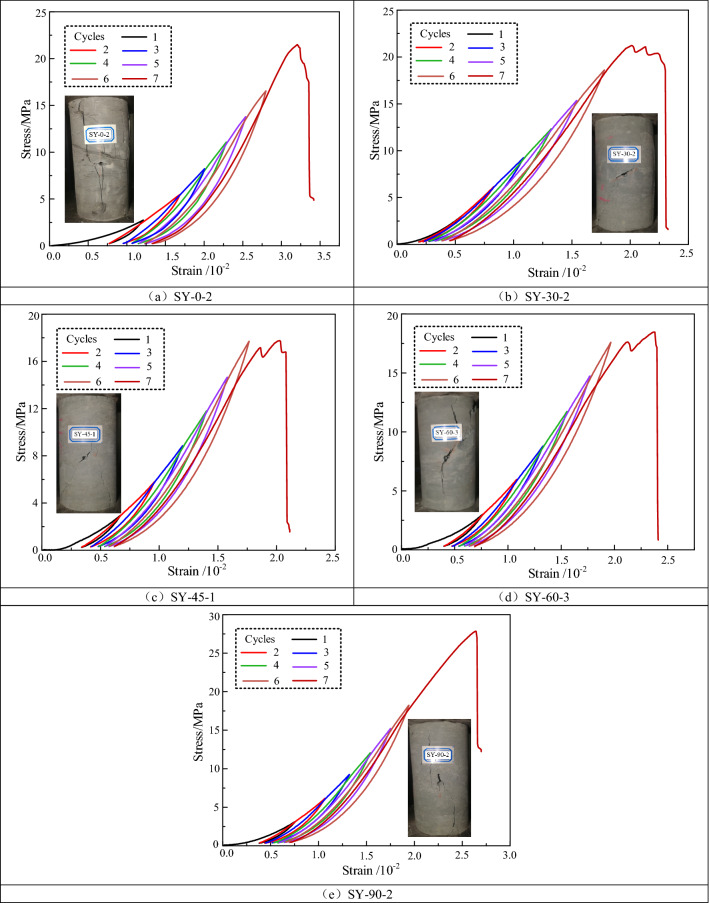
Stress–strain curves of fractured specimens under cyclic loading and unloading conditions.

Figure 3 shows the stress–strain curves of the fractured specimens under cyclic loading and unloading conditions. When the prefabricated fissure angle was 0° or 30°, the stress–strain curves had a longer compressive-density phase and a shorter elastic-deformation phase. When the prefabricated fissure inclination was 45° or 60°, the stress–strain curves exhibited a stress drop in the peak region. When the crack dip angle was 90°, the stress–strain curves dropped rapidly after reaching their peak. The stress–strain curves of the cyclic loading and unloading generally fell rapidly to lower stresses in the post-peak damage phase, indicating that after several cycles of loading and unloading, the damage was formed inside the specimens, leading to a reduction in the load-carrying capacity.

**Figure 4:**
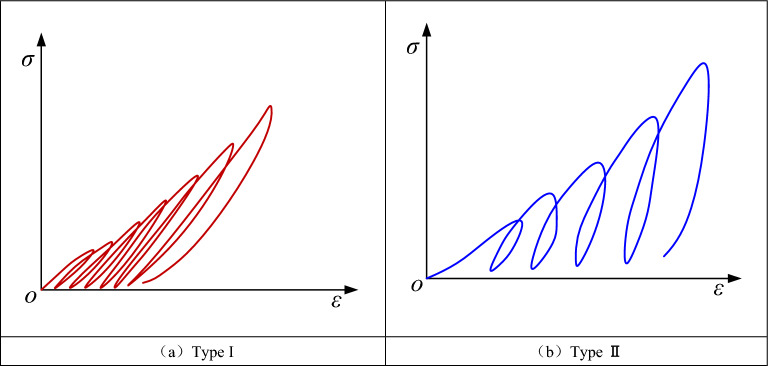
Types of stress–strain curves under cyclic loading and unloading conditions.

Figure 4 shows the types of stress–strain curves for the cyclic loading and unloading conditions. Two types of curves, Type I and Type II, were classified according to the slopes of the unloading curves. Compared with the Type I curve, the slope of the unloading curve of the Type II curve was larger, indicating that more plastic deformation occurred in the rocks during the loading process. When the inclination of the prefabricated fissure was small, the curve formed was close to the Type II curve, and as the inclination of the fissure increased, the curve is close to Type I. This was related to the angle between the fissure inclination and the loading direction. When the fissure inclination was small, the normal direction of the prefabricated fissure angle was closer to the main stress direction, and the specimens were more likely to produce plastic deformation and present a Type II curve.

### Mechanical property characteristics

**Figure 5:**
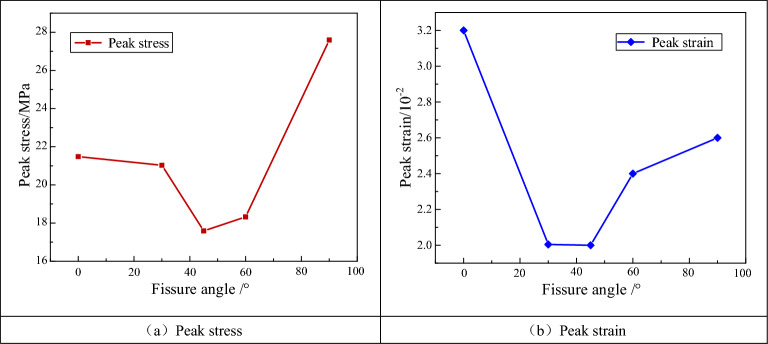
Peak stresses and strains of the fractured specimens with respect to the fracture dip angle.

Figure 5 shows the peak stresses and strains of the fractured specimens under cyclic loading conditions with respect to the fracture dip angle. And the corresponding data is shown in Table 2. As shown in Fig. 5a, the peak strengths of the specimens decreased and then increased as the prefabricated fissure angle increased. The minimum and maximum peak strengths of the specimens were 17.6 MPa and 27.9 MPa when the fracture inclination angles were 45° and 90°, respectively. As shown in Fig. 5b, the peak strains of the specimens first decreased and then increased as the prefabricated fissure angle increased. When the fracture dip angle was 0°, the maximum peak strain of the specimens was 3.2%. When the prefabricated fissure angle was 45°, the minimum peak strain of the specimens was 2.0%.

**Table 2:** Relationship between peak stress and peak strain and fracture angle of fracture specimen.

Fissure angle/°	Peak stress/MPa	Peak strain/%
0	21.48	3.20
30	21.03	2.03
45	17.59	2.01
60	18.32	2.41
90	27.59	2.62

**Figure 6:**
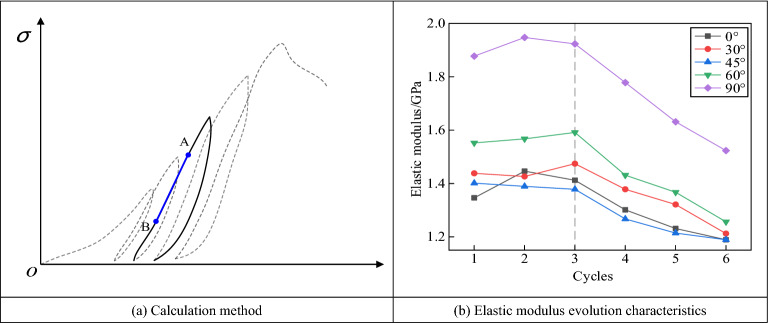
Changes of elastic modulus of fractured sandstone under cyclic loading and unloading conditions.

Figure 6 shows the calculation method for the rock elastic modulus and its variation law under cyclic loading and unloading conditions. The dashed line in Fig. 6b represents the stress–strain curve for the cyclic loading and unloading of the specimens, and the solid line represents the cyclic loading and unloading of the specimens in one round. For a certain round of cyclic loading and unloading, the linear segment of the loading phase was used to calculate the elastic modulus, that is, line segment AB in the figure.

Figure 6b shows the evolution of the elastic modulus of the fractured rocks. With an increase in cyclic loading and unloading, the elastic moduli of the fractured specimens initially increased and then decreased. During the early stage of loading, the original fissure pores of the specimens were closed, and the elastic moduli increased. As the number of cycles increased, numerous new cracks developed inside the specimens, resulting in a gradual decrease in the modulus of elasticity. Using a specimen with a prefabricated fissure angle of 90° as an example, the modulus of elasticity initially increased from 1.877 to 1.923 GPa when the number of loading and unloading cycles increased from 1 to 3. When the number of cycles was 4, the modulus of elasticity of the specimen was reduced to 1.778 GPa. The modulus of elasticity decreased to a minimum value of 1.523 GPa when the number of cycles was 6.

### Failure mode

**Figure 7:**
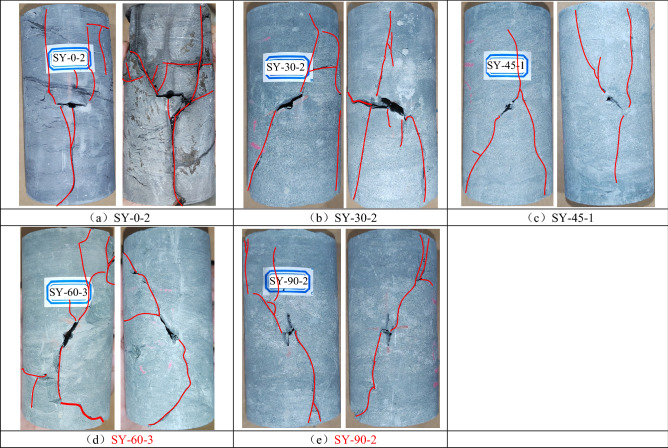
Failure patterns of the fractured specimens under cyclic loading and unloading conditions.

Figure 7 shows the failure pattern of the fractured specimens under cyclic loading and unloading conditions. As shown in Fig. 7a, when the fissure angle was 0°, a wing-shaped crack developed on the left side of the precast crack and extended upward to the top of the specimen, whereas a tensile crack developed in the middle of the precast crack and extended downward to the bottom of the specimen. As shown in Fig. 7b, when the prefabricated fissure inclination was 30°, a wing-shaped crack developed above the prefabricated fissure and extended upward over a certain distance, penetrating a tension crack, whereas a wing-shaped crack developed below the prefabricated fissure and finally extended to the bottom end of the specimen. That is, when the prefabricated fissure inclinations were 0° and 30°, the specimens were dominated by shear damage.

As shown in Fig. 7c, when the prefabricated fissure inclination was 45°, wing-shaped cracks developed above the prefabricated fissure and extended upward to the top of the specimen, whereas wing-shaped and anti-wing-shaped cracks developed below the prefabricated fracture and extended downward to the bottom of the specimen. This means that when the prefabricated fissure inclination was 45°, the specimen was in the form of tension-shear compound damage.

As shown in Fig. 7d,e, when the prefabricated fissure inclination was 60°, multiple wing-shaped cracks developed above the prefabricated fissure, whereas one wing-shaped crack developed below the prefabricated fracture. When the prefabricated fissure inclination was 90°, a crack developed in the middle of the prefabricated fissure obliquely downward, while a wing-shaped crack developed at the bottom of the prefabricated fracture and extended downward to the bottom of the specimen. It can be observed that when the prefabricated fissure inclination angles were 60° and 90°, the specimens mainly produce tensile damage.

It can be observed that when the prefabricated fissure angle was small, the crack development pattern was more complex, and a large number of shear cracks developed around the prefabricated fissure. When the prefabricated fissure inclination was larger, the crack development pattern was simpler, and the specimens were mainly damaged under tension. The above analysis shows that the damage patterns of the specimens changed from shear to tensile as the dip angle of the prefabricated fissure increased.

## Energy evolution characteristics

### Energy calculation method

The loading process of a specimen is often accompanied by an energy input, accumulation, dissipation, and release. When damping consumption and heat exchange conditions are not considered, the energy input mainly originates from the work done by the test machine on the specimen. A portion of the input energy accumulates inside the specimen in the form of elastic deformation energy, which can be released during unloading. The other part is dissipated in the form of plastic deformation and damage energies. The sum of the dissipated and elastic energies is the input energy. When the accumulation of elastic deformation energy exceeds the energy storage limit of the specimen, the specimen is damaged.

**Figure 8:**
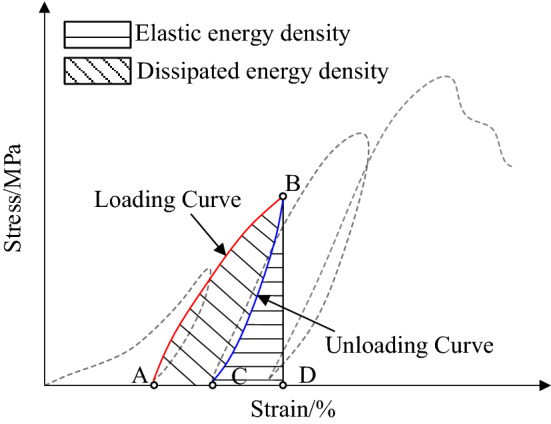
Energy calculation method under loading and unloading conditions.

Figure 8 shows the energy calculation method used during cyclic loading and unloading. In the cyclic loading and unloading processes, the red curve AB is the loading curve, and the blue curve BC is the unloading curve. The area under the red curve AB is the input energy *U* of the specimen in the loading and unloading cycle, and the area under the blue curve BC is the elastic energy *U*_e_. The dissipation energy *U*_d_ was obtained by subtracting the elastic energy from the input energy.

Based on the above energy calculation method, the input energy *U*, elastic energy *U*_e_, and dissipation energy *U*_d_ for each cycle were calculated using Eqs. (1)–(3).

1 $$U = \int_{A}^{B} {\sigma d\varepsilon }$$

2 $$U_{e} = \int_{C}^{B} {\sigma d\varepsilon }$$

3 $$U_{d} = U - U_{e}$$

### Energy evolution characteristics

**Figure 9:**
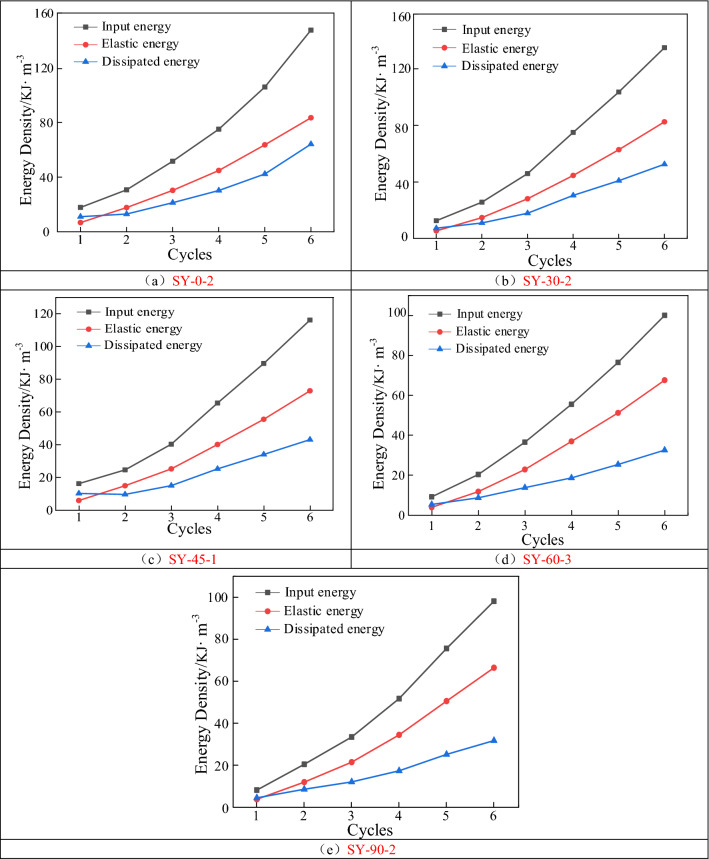
Variation curve of energy of the fractured specimens with the number of cycles.

Figure 9 shows the variation curve of the fracture specimen energy with respect to the number of cycles. As can be observed in the figure, the input, elastic, and dissipation energy densities of the fractured specimens exhibited nonlinear growth trends with an increase in the number of cycles, which can be fitted by an exponential function. At the beginning of the cyclic loading, the growth rates of the input, elastic, and dissipative energy densities were small, and the ratio of the dissipative energy density to the input energy density was large at this time. This indicates that only a small portion of the input energy was stored inside the specimens as elastic energy, and most of the input energy was used for the closure of the primary fractures inside the specimens. Using specimen SY-0-2 as an example, in the first cycle of loading, the input, elastic, and dissipative energy densities were 17.8, 6.76, and 11.04 kJ m^−3^, respectively, which indicates that most of the input energy was converted into dissipative energy. During the intermediate phase of the cyclic loading, the three types of energy densities increased approximately linearly. During the later stages of the cyclic loading, the dissipative energy density increased significantly. For example, when the number of cycles for specimen SY-0-2 increased from 5 to 6, the dissipation energy increased from 105.9 to 147.5 kJ m^−3^, with a percentage increase of 39.28%.

### Energy evolution under the influence of fissure angle

**Figure 10:**
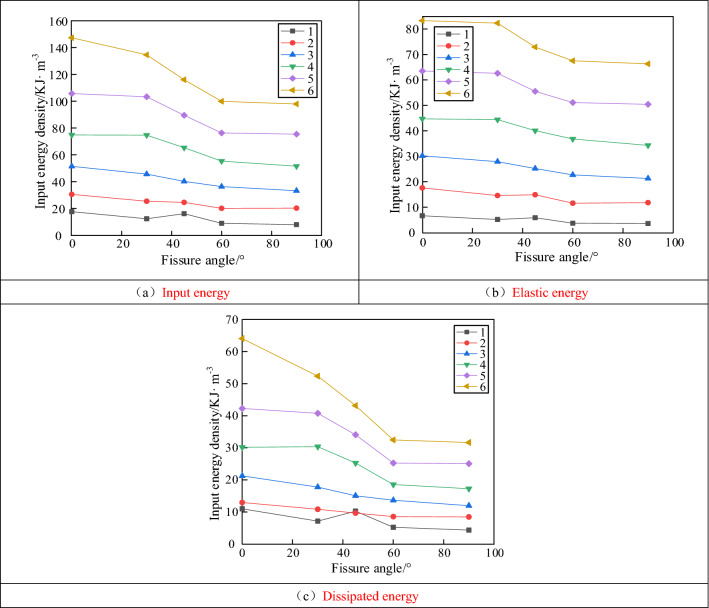
Variation curve of energy with fracture inclination under cyclic loading.

Figure 10 shows the variation curve of the fracture specimen energy with the fissure angle under cyclic loading conditions. Figure 10a shows the evolution of the input energy density of the fractured specimens. Overall, the input energy density gradually decreased as the dip angle of the prefabricated fissure increased. When the number of cycles was 1, the input energy density of the specimen reached its maximum value of 16.3 kJ m^−3^ at an angle of 45°. As the number of cycles increased, the input energy density reached its maximum of 147.5 kJ m^−3^ at an angle of 0°.

Figure 10b shows the evolution of the elastic energy density of the fractured specimens. As the prefabricated fissure angle increased, the elastic energy density gradually decreased, and the trend was similar to that of the input energy. The elastic energy evolution law exhibited a clear dip angle correlation. At the same number of loading and unloading cycles, a significant decrease in elastic energy occurred when the inclination angle increased from 30° to 60°, and the curve decreased approximately linearly. When the inclination angle increased from 0° to 30° and from 60° to 90°, the elastic energy did not change significantly.

Figure 10c shows the dissipative energy density evolution law of the fractured specimens. For the same number of cycles, the dissipated energy density gradually decreased as the prefabricated fissure angle increased. The dissipation energy curve at different inclination angles changed from a linear decreasing trend to a segmental decreasing trend as the number of loading–unloading cycles increased. The dissipative energy density reached its maximum of 64.1 kJ m^−3^ when the fissure angle was 0°. With an increase in the number of cycles, the dissipation energy density curves at different fracture dips exhibited a characteristic of "slowly decreasing-rapidly decreasing-slowly decreasing." Compared with the dissipated energy of the fifth cyclic loading, the energy of the sixth loading and unloading was significantly enhanced, and the enhancement was most significant when the fracture dip angle was 0°.

## Discussion

A large number of microfractures exist inside the rocks, and the expansion characteristics of these microfractures significantly influence the macroscopic mechanical properties of the rocks during the rock-loading process. The microfractures within the rocks generally conform to a random distribution, and these microfractures exhibit different fracture extension mechanical properties under different stress conditions. When the external load reaches the condition of fracture extension of the microcracks, the microcracks begin to extend. For fractured specimens with different fissure angles, the microfracture expansion angles under uniaxial compressive loading conditions (assuming that they are considered planar problems) are in accordance with Eq. (4).

4 $$\alpha_{{\text{i}}} + \beta_{{\text{i}}} = {9}0^\circ$$

where *α*_*i*_ is the dip angle of the microfracture, and *β*_*i*_ is the extension angle of the microfracture.

**Figure 11:**
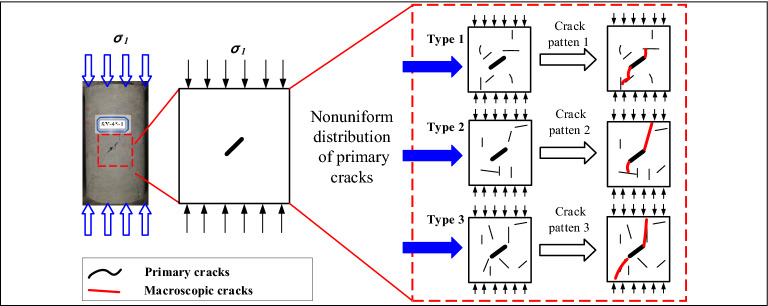
Expansion pattern of prefabricated fissures under the influence of primary cracks.

Figure 11 shows the expansion pattern of the prefabricated fissure under the influence of primary cracks. When a compression load was applied to the fractured rock specimens, stress concentration first occurred around the tips of the flaw. When the stress concentration exceeded the local strength of the material, numerous microcracks were generated around the flaw. As the compressive load increased, the microcracking activity increased and gradually clustered into macroscopic cracks. With increasing stress, the macroscopic cracks expanded along the direction of maximum principal stress, that is, the uniaxial loading direction. However, the extension of the macroscopic crack differed from the theoretical assumptions because the influence of primary cracks, as shown in Fig. 11, which mean that the nonuniform distribution of primary cracks in specimen has an important effect on crack propagation.

## Conclusions

1. The cyclic loading and unloading stress–strain curves of the fractured specimens fluctuated in the pre-peak phase and declined rapidly in the post-peak damage phase. The peak stresses and strains of the fractured specimens initially decreased and then increased with an increase in the fissure angle, whereas the elastic modulus first increased and then decreased. This indicates that cyclic loading and unloading had a compression-density effect on the internal primary fractures of the specimens at the beginning and a damaging effect at the end.
2. The crack initiation location occurred mainly at the tip of the prefabricated fissure and subsequently expanded along the direction of the maximum principal stress. With an increase in the fracture dip angle, the specimen was damaged from shear damage to tensile-shear composite damage and finally transformed into tensile damage.
3. The input, elastic, and dissipation energy densities of the fractured specimens showed a non-linear increase with an increase in the number of cyclic loading and unloading cycles. When the number of cyclic loading and unloading cycles was small, the input, elastic, and dissipation energy densities decreased linearly with an increase in the fracture dip angle. When the number of cycles was large, the energy density decreased in segments with an increase in the fracture dip, and the dissipation energy density increased rapidly prior to the damage.

## Data Availability

The datasets used during the current study available from the corresponding author on reasonable request.
